# Systematic Evaluation of the Diagnostic and Prognostic Significance of Competitive Endogenous RNA Networks in Prostate Cancer

**DOI:** 10.3389/fgene.2020.00785

**Published:** 2020-07-30

**Authors:** Zihu Guo, Liang Han, Yingxue Fu, Ziyin Wu, Yaohua Ma, Yueping Li, Haiqing Wang, Li Jiang, Shengnan Liang, Zhenzhong Wang, Furong Li, Wei Xiao, Jingbo Wang, Yonghua Wang

**Affiliations:** ^1^College of Life Science, Northwest A&F University, Yangling, China; ^2^College of Life Science, Northwest University, Xi’an, China; ^3^Department of Andrology, Fangshan Hospital, Beijing University of Chinese Medicine, Beijing, China; ^4^State Key Laboratory of New-Tech for Chinese Medicine Pharmaceutical Process, Lianyungang, China; ^5^Key Laboratory of Xinjiang Phytomedicine Resource and Utilization, Ministry of Education, Shihezi University, Shihezi, China; ^6^School of Chemistry and Pharmacy, Northwest A&F University, Yangling, China; ^7^Translational Medicine Collaborative Innovation Center, The Second Clinical Medical College (Shenzhen People’s Hospital), Integrated Chinese and Western Medicine Postdoctoral Research Station, Jinan University, Shenzhen, China

**Keywords:** prostate cancer, ceRNA network, diagnostic model, prognosis, bioinformatics analysis

## Abstract

Long non-coding RNA (lncRNA)-mediated competitive endogenous RNA (ceRNA) networks act as essential mechanisms in tumor initiation and progression, but their diagnostic and prognostic significance in prostate cancer (PCa) remains poorly understood. Presently, using the RNA expression data derived from multiple independent PCa-related studies, we constructed a high confidence and PCa-specific core ceRNA network by employing three lncRNA-gene inference approaches and key node filter strategies and then established a logistic model and risk score formula to evaluate its diagnostic and prognostic values, respectively. The core ceRNA network consists of 10 nodes, all of which are significantly associated with clinical outcomes. Combination of expression of the 10 ceRNAs with a logistic model achieved AUC of ROC and PR curve up to ∼96 and 99% in excluding normal prostate samples, respectively. Additionally, a risk score formula constructed with the ceRNAs exhibited significant association with disease-free survival. More importantly, utilizing the expression of RNAs in the core ceRNA network as a molecular signature, the TCGA-PRAD cohort was divided into four novel clinically relevant subgroups with distinct expression patterns, highlighting a feasible way for improving patient stratification in the future. Overall, we constructed a PCa-specific core ceRNA network, which provides diagnostic and prognostic value.

## Introduction

As the most prevalent malignancy among men, prostate cancer (PCa) accounted for nearly 9.1% of all male cancer deaths in 2018, making it the fourth leading cause of cancer death in Americans (Siegel et al., 2018). To date, a complete understanding of PCa initiation and progression remains elusive because its pathogenesis involves an interplay among multiple risk factors (Henrik, 2003; Bostwick et al., 2004), such as age, genetics, lifestyle, etc. While the 5-year survival rate of early PCa patients is nearly 100% owing to the improvement in modern medications, those at the advanced stage have a 5-year survival rate of less than 30% (Yuk and Kwak, 2018). Moreover, PCa patients at the advanced stage are always subject to great suffering, such as difficulty urinating and hematuria, which are usually refractory to treatment. Thus, there is an urgent need for the development of clinically relevant biomarkers for early detection and prognosis prediction of PCa, which will increase the chances for effective treatment and improve our understanding of the underlying mechanisms.

In recent years, increasing evidence has demonstrated the critical role of the interactions among endogenous RNA, namely, the competitive endogenous RNA (ceRNA) network (Salmena et al., 2011), in the development of complex disease phenotypes, including cancers (Karreth et al., 2011; Xia et al., 2019). In the ceRNA networks, miRNAs, which are a class of non-coding RNA transcripts with 20–22 nucleotides (Ambros, 2004), inhibit the expression of target genes and long non-coding RNAs (lncRNAs), leading to lncRNA-gene associations by competitively binding to shared miRNAs. LncRNA, which has been reported to be closely involved in cancer occurrence and progression (Zhan et al., 2018; Hua et al., 2019), is defined as a type of RNAs without coding potential, which are more than 200 nucleotides in length (Perkel, 2013; Zampetaki et al., 2018).

Therefore, construction of lncRNA-modulated ceRNA networks may have potential for identifying candidate biomarkers and has been investigated in multiple human cancers. For example, Fang et al. constructed a ceRNA network associated with head and neck squamous cell carcinoma (HNSCC) and further identified a series of RNAs exhibiting a significant impact on overall survival (Fang et al., 2018). By establishing an mRNA-miRNA-lncRNA subnetwork for pancreatic cancer, Wang et al. demonstrated an association between the ceRNA network and prognosis of pancreatic cancer (Wang et al., 2019). Thus, the ceRNA network may also present a promising strategy for potential biomarker identification for PCa. Recently, several independent studies (Jiang et al., 2018; Xu et al., 2018; Li F. et al., 2019; Ye et al., 2019) identified a handful of prognostic biomarkers for PCa based on lncRNA-mediated ceRNA networks. For example, Liu et al. proposed a systems biology approach to infer the gain and loss of ceRNAs and identified several competitive RNA pairs showing prognostic values (Liu et al., 2016), which proved to be useful for developing therapeutic regimens. Xu et al. constructed a ceRNA network comprised 94 PCa-specific ceRNAs, which may play a critical role in the progression and metastasis of PCa (Xu et al., 2018). These studies promote our understanding in developing novel diagnostic and prognostic biomarkers for PCa. Nevertheless, systematically elucidating the diagnostic and prognostic value of the ceRNA network in PCa is still insufficient. First, most previous studies were conducted based on single dataset, leading to an increase of the risk of data bias due to technical and biological noise. In addition, diagnostic and prognostic related biomarkers were usually identified independently, which may hinder our understanding of the relative complete process from PCa initiation to deterioration. What’s more, the application potential of ceRNA network on other clinical prognosis related problems, such as molecular based PCa patient’s stratification, still remains unclear. Therefore, presently, we aim to compressively investigate the diagnostic and prognostic value of the ceRNA network in PCa based on multiple independent datasets.

In the present work, we initially integrated dysregulated RNAs derived from multiple independent PCa-related studies to make this analysis more reliable. Functional enrichment analysis on the commonly appearing genes was subsequently performed to investigate the underlying mechanism. Then, a high-confidence and PCa-specific ceRNA network was constructed using the integrated computational tool GDCRNATools (Li R. et al., 2018) based on three different lncRNA-gene association inference strategies. The nodes with significant clinical outcomes were thereby screening to construct the core ceRNA architecture. Afterward, using the combination of expression of RNAs in this core ceRNA network, a logistic model and risk score formula were established to evaluate its diagnostic and prognostic values, respectively. Finally, using an unsupervised clustering approach, we further investigated whether the ceRNA network-based expression profile has the ability to define novel molecular subtypes with prognostic information.

## Materials and Methods

### RNA Expression Profiles and Clinical Data Retrieval

In order to identify the endogenous RNAs (ceRNAs) potentially involved in the initiation and development of PCa, expression profiles of several PCa-related studies were taken into account. In the first step, miRNA expression profiles of two independent, PCa-related studies were downloaded from the Gene Expression Omnibus (GEO) database (Edgar et al., 2002) (GSE76260 and GSE21036). GSE76260 includes 32 pairs of PCa tumor tissues and adjacent non-neoplastic tissues, the GSE21036 dataset contains 99 tumor tissues (the metastasis samples were not included) and 28 normal adjacent benign prostate tissue. These two datasets were generated on the Illumina Human v2 MicroRNA expression beadchip (GPL8179) and Agilent-019118 Human miRNA Microarray 2.0 G4470B (GPL8227), respectively. As for the mRNA and lncRNA, two RNA-seq datasets derived from independent PCa-related studies were obtained from GEO. GEO accession numbers of the two datasets are GSE89223 (*n* = 28) and GSE104131 (*n* = 32), which contain 14 and 16 PCa tumor samples, respectively. Among them, GSE89223 was based on the GPL17303 platform (Ion Torrent Proton), and GSE104131 was based on the GPL16791 platform (Illumina HiSeq 2500).

In addition to the datasets retrieved from GEO, TCGA-PRAD (Abeshouse et al., 2015) RNA-seq and miRNA sequence data plus clinical metadata were downloaded with permission from the Cancer Genomics Hub^1^ via the “TCGAbiolinks” package (Colaprico et al., 2015). This dataset contained 547 samples, including 495 PCa tumors and 52 normal tissues. The RNA-seq and miRNA sequence data for these samples were generated with the IlluminaHiSeq RNASeq and IlluminaHiSeq miRNASeq sequencing platforms, respectively. Clinical metadata of the 495 PCa patients contained their age, TNM stage, molecular subtypes, disease-free survival data and outcome.

### Identification of Differentially Expressed mRNAs, lncRNAs, and miRNAs

To obtain the differentially expressed mRNAs, lncRNAs and miRNAs, a widely used linear model, limma, was performed as previously described (Ritchie et al., 2015). Briefly, for each RNA expression dataset, the “limma” package (Ritchie et al., 2015) from Bioconductor (Gentleman et al., 2004) was applied to conduct differential expression analysis between PCa tumor samples and the normal controls. The RNAs with Log_2_(*foldchange*) > 0.26 and *p*-value <0.01 were considered significantly differentially expressed. We should note here, according to previous studies (Conway et al., 2017), this threshold value is a widely accepted differential gene screening criteria. Additionally, in this study, we aim to find the ceRNAs with generally prognostic significance in PCa from multiple independent studies. Therefore, we chose to adopt this relatively loose threshold instead of a more stringent threshold to avoid the sample selection bias. Then, the lists of differentially expressed RNAs derived from the three expression datasets were compared using matrix-based visualization created by UpSetR (Harrow et al., 2012). The gene transfer format (GTF) file for human, which contains gene type annotation (including “protein coding,” “lincRNA,” etc.), was download from the GENCODE project (Paraskevopoulou et al., 2012)^2^, and was then applied to identify the lncRNAs and mRNAs from the overlapping differentially expressed genes. The differentially expressed mRNAs and lncRNAs that commonly appeared in the three datasets were retained for further analysis. For the miRNAs, the same procedure was applied to determine differentially expressed miRNAs commonly appearing in these three miRNA expression datasets.

### Construction of a ceRNA Regulatory Network

In the present study, the construction of a ceRNA regulatory network involved retrieving miRNA-target associations from several publicly available databases as well as predicting competitive relationships between lncRNAs and mRNAs with three strategies.

First, putative interactions between differentially expressed miRNAs and lncRNAs were retrieved from the DIANA-LncBase v.2 (Li et al., 2013), starBase v2.0 (Jeggari et al., 2012), miRcode 11 (Ning et al., 2016) and lincSNP 2.0 (Wong and Wang, 2014) databases. Then, all putative interactions from the four databases were aggregated together. As a result, we obtained 7,359,063 miRNA-lncRNA pairs. For possible miRNA-gene pairs, their putative and experimentally validated interactions were retrieved from four sources: miRDB 6.0 (Hsu et al., 2010), miRTarBase 7.0 (Agarwal et al., 2015), starBase v2.0 (Jeggari et al., 2012) and TargetScan 7.2 (Shannon et al., 2003). After aggregating these interactions, we obtained a total of 98,436,283 miRNA-mRNA associations.

Next, we dissected the competitive associations between the differentially expressed mRNAs and lncRNAs using the R/Bioconductor package “GDCRNATools” (Li R. et al., 2018), which determines the competing endogenous interactions based on three criteria: (1) the number of miRNAs shared by gene and lncRNA must be significant (hypergeometric test, *p*-value <0.05); (2) in this cohort, there must be a positive correlation between the expression of gene and lncRNA (*R*^2^ > 0, *p*-value <0.05); and (3) those shared miRNAs by lncRNA and gene should play similar regulatory roles according to a recently defined regulation similarity score (Li R. et al., 2018) (regulation similarity score >0). Thus, mRNA-lncRNA associations that satisfied these three criteria were considered high-confidence competitive interactions, which were subsequently used for ceRNA network construction.

Finally, the miRNA-mRNA, miRNA-lncRNA and lncRNA-mRNA interactions were integrated and imported into Cytoscape software (Yu et al., 2012) (V 3.6.0) for ceRNA network visualization.

### Identification of the Core Competitive Endogenous RNA Network

In order to extract the clinically significant core ceRNA architecture from the primary ceRNA network constructed above, three criteria were applied to assess the association between each node and the clinical trait. First, using the RNA expression profile and clinical data in TCGA, for each node in the above network, the effectiveness in distinguishing the tumor and normal samples was evaluated by the area under the curve (AUC) of the receiver operating characteristic (ROC) curve. The nodes with a high AUC (>0.80) were regarded as candidate diagnostic RNAs, which were retained for further analysis. In addition, we also evaluated the extent to which RNA expression was associated with disease-free survival using both the log-rank test and Cox regression (see survival analysis in methods for details). Specifically, based on the PCa patients’ disease-free survival data in TCGA, the prognostic effect of 208 RNAs was initially assessed with Kaplan–Meier analysis. The Benjamini & Hochberg procedure was applied for false discovery rates (FDR) calculation to adjust p-values. The nodes with a log-rank test FDR < 0.1 (*p*-value <0.05) were considered statistically significant. Additionally, Cox-proportional hazards regression, adjusting for clinical parameters such as age at diagnosis and TNM stage, was performed to further identify the RNAs with prognostic significance. We set the criteria as FDR < 0.25 (*p*-value <0.05) to identify candidate nodes.

Finally, the common RNAs that satisfied the three criteria were treated as significantly diagnostic and prognostic candidate nodes, which were subsequently mapped to the primary ceRNA network. As a result, only the interactions between the candidate RNAs were retained, and the final refined ceRNA network, namely, the core ceRNA network, was also visualized with Cytoscape software (Yu et al., 2012).

### Functional Analysis of the Differentially Expressed mRNAs

To investigate potential molecular mechanisms involved in PCa development, the Gene Ontology biological process (GOBP) and Kyoto Encyclopedia of Genes and Genomes (KEGG) pathway enrichment analyses were performed via the “clusterProfiler” package (Bishop, 2006). The GOBP and KEGG enrichment analyses were conducted based on the up regulated and down regulated mRNAs, respectively, and the significant cut-off criteria were set as *p*-values <0.05. For visualization, the top 15 most significant GOBP and KEGG terms are exhibited as bubble charts and bar charts, respectively.

### Diagnostic Model Construction and Performance Evaluation

To better understand the diagnostic significance of the ceRNA network, using the TCGA-PRAD dataset, a logistic regression model was built based on the combination of the expression levels of the RNAs in the ceRNA network. The logistic model, which is a widely used model for supervised machine learning (Therneau and Lumley, 2014), was adopted to discriminate the PCa tumor samples from the normal samples. It was established with the following formula:

$$y=\frac{1}{1+\exp\left(-\beta_{0}-\sum \alpha_{i}\times E_{i}\right)}$$

where *y* is the probability of the tissue being from the tumor sample, β_*0*_ is the y-intercept, and α_*i*_ is the coefficient of a given RNA expression *E*_*i*_. The coefficients were estimated using maximum-likelihood estimation approach. To avoid overfitting, leave one out cross validation (LOOCV) was applied to assess the performance of this model based on the TCGA-PRAD dataset. Specifically, one sample was taken from the entire TCGA-PRAD dataset and was used as the test data, and the remaining samples were used as training data. This process was repeated 547 times, and the prediction was stored at each step to evaluate the final performance of the diagnostic model. The AUC values of the receiver operating characteristic curve (ROC) and Precision-Recall (PR) curves were calculated with the ROCR package. What’s more, the diagnostic value of individual RNA in the ceNRA network was also assessed by AUC of PR curves using ROCR package.

To further validate the diagnostic value of the core ceRNA network, we compared its AUC of ROC and PR with the result obtained from randomly selected gene sets, which was served as a background. Beefily, the same number of RNAs as the core ceRNA network were randomly selected from the expression profile, and were used to constructed a diagnostic model using the logistic regression model as described above. The AUC values of the ROC and PR curve were calculated with the ROCR package. This procedure was repeated 1,000 times to generate distributions of both the AUC values of the ROC and PR curve.

### Survival Analysis

For each RNA in the ceRNA network, we performed both univariate Cox-proportional hazards regression and Kaplan–Meier analysis to evaluate its prognostic value. The univariate Cox-proportional hazards regression model, which adjusted for clinical parameters such as age at diagnosis and TNM stage, was built to determine the relationship between the expression level of each RNA and PCa patient disease-free survival retrieved from TCGA. It was established with the “survival” package (Kassambara et al., 2017), and the coefficient and hazard ratio of each RNA were also calculated. The Benjamini & Hochberg procedure was applied for FDR calculation to adjust *p*-values. FDR <0.25 (*p*-value <0.05) for the Cox regression was considered as the statistically significant cut-off. Additionally, for each RNA, the Kaplan–Meier analysis with a non-parametric log-rank test was used to compare the disease-free survival of the PCa patients in the low- and high-expression groups. The “survminer” package (Chen et al., 2019) was used for Kaplan–Meier plot generation and log-rank test p-value calculation. In addition, those RNAs with a log-rank test *p*-value <0.05 were regarded as candidate prognosis biomarkers.

### Prognostic Risk Score Model Construction and Assessment

To further assess the prognostic significance of the core ceRNA network, a prognostic risk score model was constructed as described previously (Van der Laan et al., 2003; Liu et al., 2018). First, the univariate Cox regression analysis was performed on each RNA using the TCGA-PRAD chart as described above. The coefficient for each RNA on the core ceRNA network was extracted for further construction of the prognostic risk score model. Then, the risk score model was constructed based on the linear combination of the core ceRNAs’ expression levels:

$$\mathrm{risk}\,\mathrm{score}=\sum_{i=1}^{N}\gamma_{i}\times E_{i}$$

where γ_*i*_ is the coefficient of RNA *i* obtained from Cox regression, *E*_*i*_ is the expression of RNA *i*, and *N* is the number of RNAs in the core ceRNA network. Finally, for each patient, their risk score was calculated based on the expression data of the selected RNAs. According to the median cut-off of the risk scores, the TCGA-PRAD patients were divided into high-risk and low-risk subgroups for further analysis. The association between the risk score was assessed with Fisher’s exact test, and a *p*-value <0.05 was considered the significant cut-off.

### Patient Stratification Based on the Core ceRNA Network

In order to further investigate whether using the core ceRNA network is capable of dividing the entire cohort into clinically relevant subgroups, unsupervised cluster analysis was performed with a standard consensus clustering framework. First, RNA expression in the TCGA-PRAD cohort was normalized by a z-score transformation. This was only performed on the RNAs of the core ceRNA network. The z score of each RNA was calculated as follows:

$$\mathrm{score}\,\left(G_{i}\right)=\frac{G_{i}-\mu \,\left(G\right)}{\delta \,\left(G\right)}$$

where *G*_*i*_ is the expression level of RNA *G* in patient *i*, and μ(*G*) and δ(*G*) are the mean expression level and standard deviation of RNA *G* across all samples, respectively. Then, we computed Pearson’s correlation coefficient (PCC) between samples to assess their similarity and subsequently divided the samples into *k* subgroups using the partition around medoids algorithm (Wilkerson and Hayes, 2010). The maximizing cluster reliability approach was adopted to determine the optimal number of subgroups as described previously. Consensus clustering analysis was implemented with the “ConsensusClusterPlus” package (Wickham, 2011).

To further validate and visualize core ceRNA network-derived clustering, principal component analysis (PCA) was performed among the TCGA-PRAD tumor samples. The PCA analysis was conducted based on the normalized expression data using the “prcomp” function from the “stats” module in the R 3.3.6 system. We then selected the first two principal components with the most variance to project each sample into two-dimensional space. Finally, the expression pattern of all samples as well as the core ceRNA network-derived clusters was visualized with the “ggplot2” package (Raspe et al., 2012).

## Results

### Identification of PCa-Associated mRNAs, miRNAs, and lncRNAs

Due to the poor reproducibility across biological replicates and multiple studies, the RNA expression profile has not been broadly adopted in clinical tests (Hanahan and Weinberg, 2011; Alvarez et al., 2016). Thus, to enhance the reliability of this study, we integrated PCa-related mRNAs, miRNAs and lncRNAs derived from multiple independent traits.

**FIGURE 1:**
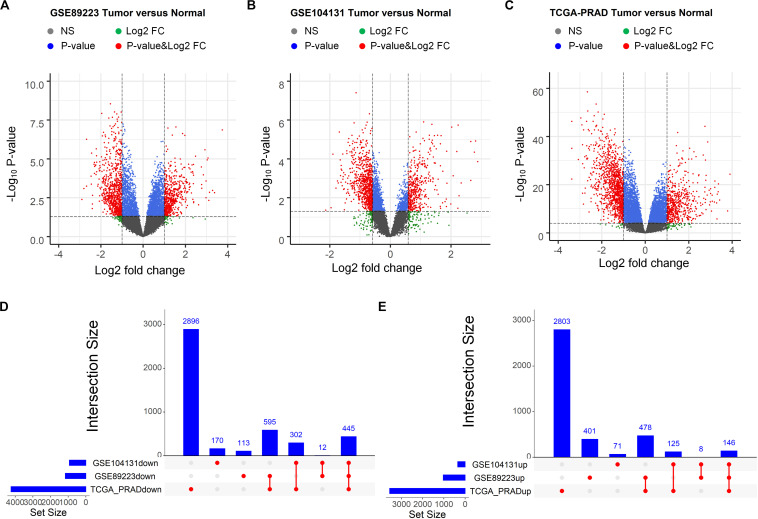
Identification of differentially expressed mRNAs between prostate tumor tissue and normal control. **(A–C)** Volcano plot of differentially expressed mRNAs derived from three independent PCa related studies: GSE89223 **(A)**, GSE104131 **(B)** and TCGA-PRAD **(C)**. Red spots represent significantly deregulated mRNAs. **(D,E)** UpSet plot of overlapping up regulated and down regulated mRNAs from three independent PCa related studies.

First, RNA-seq profiles as well as their corresponding clinical information of three PCa-related studies were retrieved, of which two were downloaded from the GEO (Edgar et al., 2002) and one was downloaded from TCGA (Abeshouse et al., 2015). The “limma” package (Ritchie et al., 2015) was then applied to identify the differentially expressed RNAs by comparing the expression profiles between the normal tissues and tumor tissues (Methods). As a result, 1,033, 350 and 3,552 up regulated genes as well as 1,165, 929 and 4,238 down regulated genes were obtained from the three datasets, respectively (Figures 1A–C). As showed in Figures 1D,E, 146 up regulated and 445 down regulated genes commonly appeared in the three datasets. We then used the gene transfer format (GTF) file for human retrieved from GENCODE project (Paraskevopoulou et al., 2012) to identify the lncRNAs and mRNAs from the overlapping differentially expressed genes. We finally obtained 21 up regulated and 15 down regulated lncRNAs, as well as 125 up regulated and 430 down regulated mRNAs.

**FIGURE 2:**
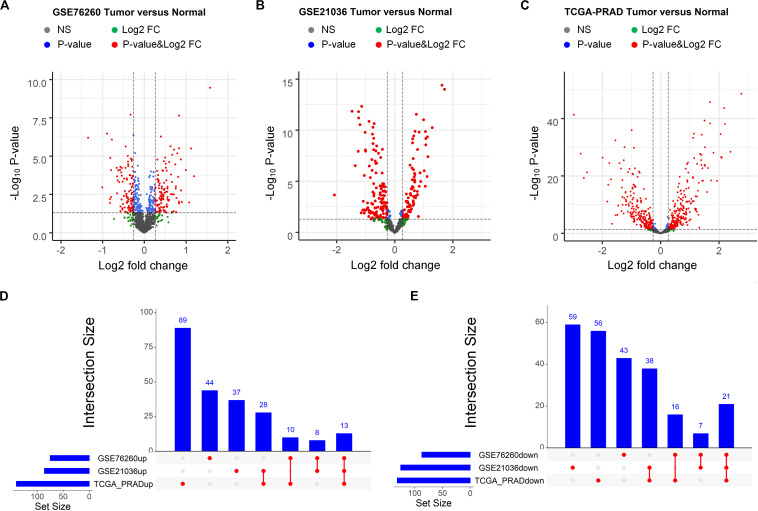
Identification of differentially expressed miRNAs between prostate tumor tissue and normal control. **(A–C)** Volcano plot of differentially expressed miRNAs derived from three independent PCa related studies: GSE76260 **(A)**, GSE21036 **(B)** and TCGA-PRAD **(C)**. Red spots represent significantly deregulated miRNAs. **(D,E)** UpSet plot of overlapping up regulated and down regulated miRNAs from three independent PCa related studies.

Then, the same procedure was conducted to identify the miRNAs that potentially contribute to PCa occurrence. We initially downloaded two miRNA expression profiles and the corresponding clinical information from the GEO and one from TCGA. Note that the three miRNA expression profiles are derived from three independent PCa-related studies. Afterward, as showing in the volcano plots (Figures 2A–C), the differential analysis performed by “limma” on the three datasets allowed the identification of three lists of deregulated miRNAs in PCa tissues compared to that in normal controls. By comparing these three lists of miRNAs, we identified 35 miRNAs that were differentially expressed in all three datasets, 13 of which were up regulated and 21 of which were down regulated in the tumor tissues (Figures 2D,E). The overlapping differentially expressed miRNAs as well as the mRNAs and lncRNAs may be strongly associated with the presence of PCa. Thus, they were selected for subsequent analyses.

### GO and Pathway Enrichment Analyses for the PCa-Associated mRNAs

**FIGURE 3:**
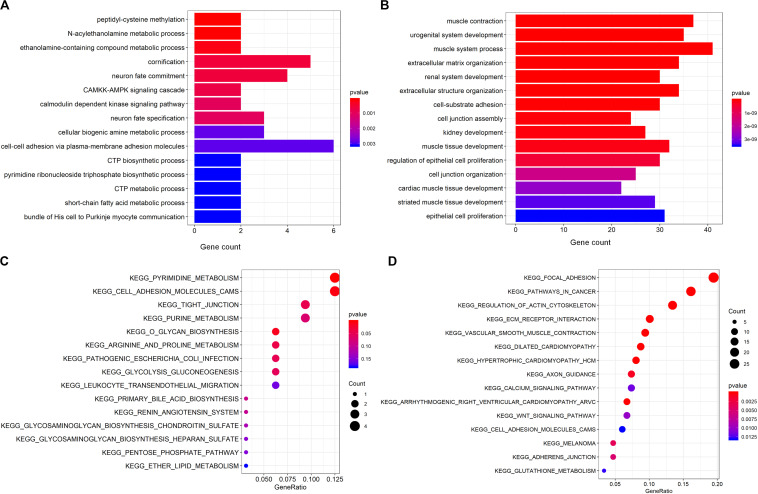
GOBP and KEGG enrichment on the commonly appeared differentially expressed genes. **(A,B)** GOBP enrichment analysis on the commonly up regulated genes and down regulated genes. The *y*-axis is GOBP term, and the *x*-axis is the number of genes enriched to the corresponding term. **(C,D)** KEGG pathway enrichment analysis on the commonly up regulated genes and down regulated genes. The *y*-axis is KEGG pathway term, and the *x*-axis is the gene ratio representing the proportion of enriched genes in a KEGG pathway term over the number of genes in the inputted gene list.

In order to explore the functional roles of deregulated genes in the pathogenesis of PCa, GO biological process (GOBP) and KEGG enrichment analyses on the overlapping up regulated and down regulated mRNAs were performed using the “clusterProfiler” package (Bishop, 2006). Figures 3A,B illustrate the top 15 most significantly enriched GOBP and KEGG terms for the overlapping up regulated genes. As seen, there was a significant enrichment for metabolism process-related terms, including “*N*-acylethanolamine metabolic process,” “CTP metabolic process” and “short-chain fatty acid metabolic process.” According to the KEGG analysis, the results demonstrated that the pathways found to be mainly involved were consistent with the enriched GOBP terms. Specifically, the most significantly enriched pathways are metabolism-related pathways such as “pyrimidine metabolism,” “lipid metabolism” and “arginine and proline metabolism.” It has been well demonstrated that metabolic alteration is a hallmark of PCa initiation (Tennant et al., 2010), and serves as a novel therapeutic target for cancer treatment (Liu, 2006). For example, according to a previous study, fatty acid metabolism is a critical bioenergetic pathway in PCa, and has the potential to diagnosis and targeted treatment (Migita et al., 2009). What’s more, Migita et al. have reported that fatty acid synthase, a key regulator for the synthesis of long-chain fatty acids, is involved in PCa occurrence (Wu et al., 2014). Additionally, dysregulation of lipid metabolism such as altered enzyme activity induced lipid accumulation has been a hallmark of the malignant phenotype of PCa (Li X. et al., 2019). Recent plasma metabolic profiling revealed that as one of the most influenced metabolic pathway, arginine and proline metabolism is also highly associated with PCa. More importantly, it was demonstrated that the alteration of metabolic processes is not only a hallmark of cancer development but also a potential target for enhancing antitumor immunity (Hennenberg et al., 2014). Collectively, these results suggest that one of the main mechanisms for PCa initiation and progression is metabolic disorder.

Regarding the overlapping down regulated mRNAs, the most significantly enriched GOBP and KEGG terms can be seen from Figures 3C,D. As illustrated, these genes were mainly enriched for muscle system process-related terms such as “muscle contraction.” Hennenberg et al. reported that prostate smooth muscle contraction may be critically involved in maintaining the physiological function of lower urinary tract (Paul et al., 1997), which usually exhibits severe pathological changes in PCa patients. In addition, a significant enrichment for the cell adhesion-related process, such as “cell-substrate adhesion,” “cell junction assembly” and “cell junction organization,” is also displayed in Figure 3C. Importantly, dysfunction of the cell adhesion-related pathway is involved in PCa invasiveness and progression, and the genes in this pathway are important prognostic markers in PCa, which were reported correlated with the PCa grade, patient survival and recurrence after radical prostatectomy (Yardy and Brewster, 2005). Further dissection of these genes and KEGG pathways confirmed enrichment for cell adhesion (e.g., “focal adhesion” and “adherens junction”). What’s more, we also observed a significant enrichment in various signal transduction pathways, including the “calcium signaling pathway (map04020)” and “wnt signaling pathway (map04310).” And both of these two pathways have been proven to play critical roles in PCa development (Wasilenko et al., 1997; Albert-László et al., 2011), which may provide insight into the upstream molecular mechanism for PCa progression. Taken together, these results suggest that the overlapping deregulated genes play a crucial role in modulating different functions and pathways involved in PCa initiation and progression, implying their potential diagnostic and prognostic significance.

### Construction of the Prostate Cancer-Associated ceRNA Regulatory Network

**FIGURE 4:**
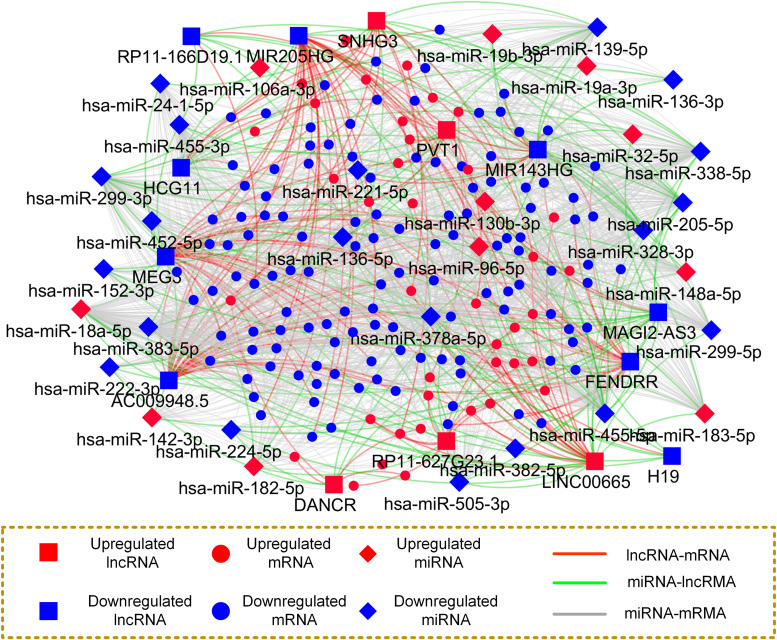
Construction of PCa tissue specific and high confidence ceRNA network. Nodes in this network represent different types of ceRNAs, and edges represent the interactions among these ceRNAs.

To dissect the regulatory relationship between the differentially expressed mRNAs, miRNAs and lncRNAs in PCa comprehensively, the ceRNA network was constructed using a R/Bioconductor package, i.e., GDCRNATools (Li R. et al., 2018). Initially, competitive associations between the lncRNA-mRNA pairs were determined based on the significance of the hypergeometric test, Pearson correlation analysis and regulation pattern analysis (Methods). The miRNA-mRNA and miRNA-lncRNA interactions were retrieved from 7 miRNA-target databases (Methods). Subsequently, we constructed a mRNA-miRNA-lncRNA network and visualized it with Cystoscope software (Yu et al., 2012) (Version 3.6.0), as showed in Figure 4.

The resulting ceRNA network consists of 207 nodes and 1,588 edges. In detail, the nodes in the network include 39, 11, and 5 up regulated as well as 123, 20, and 9 down regulated mRNAs, miRNAs and lncRNAs, respectively. Additionally, the numbers of lncRNA-mRNA pairs and miRNA-target pairs in the ceRNA network are 186 and 1,423, respectively. The average number of targets per miRNA is 44.47, indicating the multitarget regulation features of the miRNAs. For instance, the miRNA with the highest connective degree is hsa-miR-378a-5p, followed by hsa-miR-455-5p with 113 miRNA-target interactions and hsa-miR-18a-5p possessing 100 targets.

By further observation of the ceRNA network, we found that some lncRNAs competitively associated with multiple genes, implying their functional role in regulating PCa initiation via mRNAs. For example, 10 mRNAs were found potentially regulated by the lncRNA SNHG3. Interestingly, we also observed some lncRNAs compete with similar genes. For instance, more than 20% competitive partner genes of SNHG3 were also regulated by LINC00665, indicating that they may exhibit a similar mechanism in the tumor. All the findings imply that cancer initiation and progression are driven by aberrant expression of multiple types of endogenous RNAs working in concert to regulate key tumor hallmarks.

### Identification of the Nodes With Diagnostic and Prognostic Significance

The ceRNA network constructed above provides a primary regulatory landscape among the endogenous RNAs. However, because this network consists of numerous compounds and interactions among them, it is difficult to elucidate its diagnostic and prognostic significance. In order to further identify key nodes in this ceRNA network, for each individual node, we assessed its effectiveness in distinguishing the tumor and normal samples as well as to what extent it was associated with disease-free survival.

To evaluate the diagnostic accuracy of each node in the ceRNA network, the expression levels were examined in 495 PCa samples and 52 normal samples from TCGA, and the AUC of the ROC curve was subsequently calculated. We found that more than 60% of the node exhibited high AUC values (>0.80), including 111 genes, 12 miRNAs and 8 lncRNAs. This finding implied that the ceRNA network may provide a new way to identify biomarkers for the diagnosis of PCa.

We further analyzed the expression level of each node and survival data of 495 TCGA-PRAD patients to identify potential prognostic biomarkers. These patients were divided into two groups based on the normalized expression level, and the log-rank test was used to compare the differences in disease-free survival times between the two groups. We identified 140 out of 208 nodes exhibiting significant prognostic value (FDR < 0.1, *p*-value <0.05, log-rank test). Furthermore, we performed univariate Cox-proportional hazards analysis on the expression of each RNA, adjusting for clinical parameters including age at diagnosis and TNM stage. This analysis showed that 44 of these nodes were significantly associated with disease-free survival (FDR < 0.25, *p*-value <0.05).

**FIGURE 5:**
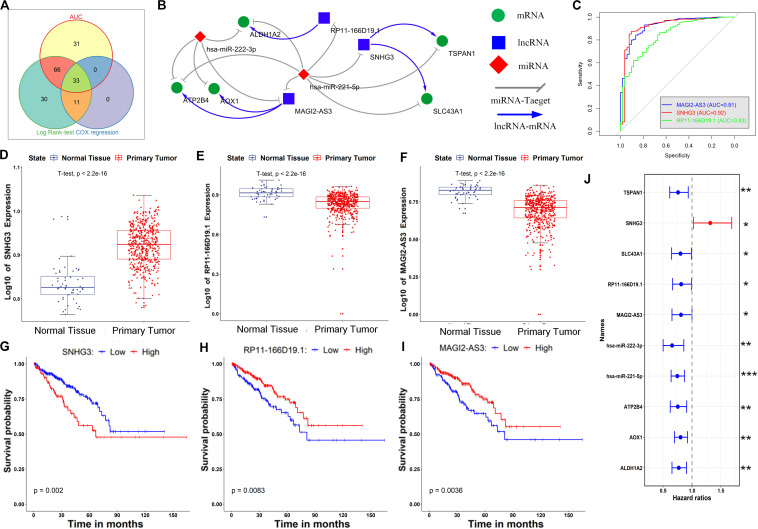
Identification of key RNAs in the ceRNA network and constructing a core ceRNA network. **(A)** Identification of key RNAs by combining diagnosis and prognosis analyses. **(B)** The core ceRNA network constructed by mapping the key RNAs to the primary ceRNA network. **(C)** The AUC of ROC for discriminating the tumor and normal tissues based on the expression of lncRNAs in the core ceRNA network. **(D–F)** Expression of SNHG3, RP11-166019.1 and MAGI2-AS3 in the normal and tumor tissues of PCa. **(G–I)** Kaplan–Meier curves of prognostic models built with the expression of SNHG3, RP11-166019.1 and MAGI2-AS3, respectively. **(J)** Forest plot of hazard ratios showing the prognostic values of all RNAs in the core ceRNA network. *p < 0.05; **p < 0.01; ***p < 0.001.

Then, the common RNAs with both high diagnostic value (AUC > 0.80) and prognostic significance (FDR < 0.25 for Cox regression and FDR < 0.25 for log-rank test) were selected as the candidate RNAs for further analysis. Thirty-three RNAs were eventually identified (Figure 5A), including 28 genes, 3 lncRNAs and 2 miRNAs (Supplementary Table S1). These RNAs can be considered candidate key regulators contributing to both the initiation and development of PCa.

### Construction of a Core ceRNA Network and Building Novel Diagnostic and Prognostic Models

It has been well established that cancer initiation and progression are a consequence of abnormalities in numerous intracellular compounds, including multiple types of RNAs, as well as interactions among them (Glinskii et al., 2011). We therefore extracted the interactions among the candidate key RNAs to construct a core ceRNA network architecture that possesses diagnostic and prognostic significance.

Initially, the 33 candidate RNAs obtained above were mapped to the primary ceRNA network, and interactions between the candidate RNAs were retained. The resulting ceRNA network, namely, the core ceRNA network, is given in Figure 5B. The core ceRNA network consists of 3 lncRNAs (SNHG3, RP11-166D19.1 and MAGI2-AS3), 2 miRNAs (hsa-miR-222-3p and hsa-miR-221-5p) and 5 genes (ATP2B4, AOX1, ALDH1A2, TSPAN1 and SLC43A1), as well as 16 edges among them. Notably, most of these ceRNAs have been shown to play a role in PCa onset or progression. For instance, the lncRNA SNHG3 is capable of driving castration-resistant phenotype of PCa (Zhou et al., 2013), and its dysregulation was predicted well associated with PCa initiation (Coarfa et al., 2015); hsa-miR-221-5p is able to suppress PCa cell proliferation by targeting key oncogenic pathways, including apoptosis, Akt/mammalian target of rapamycin signaling, metastasis and the androgen receptor (AR) axis (Yusuke et al., 2015); hsa-miR-222-3p is down regulated in PCa, and increased hsa-miR-222-3p can significantly inhibit cell migration (Li W. et al., 2018); a genome-wide scan identifies that mutation of the AOX1 locus is associated with PCa-specific survival time (Xu et al., 2016); decreased TSPAN1 promoted PCa progression and served as a biomarker for early biochemical recurrence (Hanna et al., 2005); ALDH1A2, which is a retinoic acid synthesis gene, has been previously reported as a candidate tumor suppressor in PCa (Hofree et al., 2016); and inhibition of SLC43A1 may provide a novel therapeutic target in PCa, via suppression of cell cycle genes (Xu et al., 2016). Hence, the critical role of this ceRNA network was confirmed by these independent studies.

**FIGURE 6:**
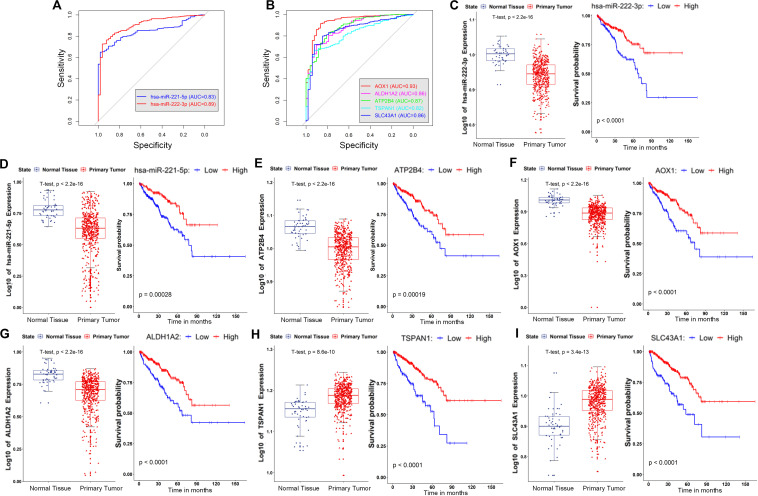
Evaluation of the diagnostic and prognostic value of the genes and miRNAs in the core ceRNA network. **(A)** The AUC of ROC for discriminating the tumor and normal tissues based on the expression of miRNAs (hsa-miR-222-3p and hsa-miR-221-5p) in the core ceRNA network. **(B)** The AUC of ROC for discriminating the tumor and normal tissues based on the expression of mRNAs (ATP2B4, AOX1, ALDH1A2, TSPAN1, and SLC43A1) in the core ceRNA network. **(C–I)** left: Expression of two miRNAs (hsa-miR-222-3p and hsa-miR-221-5p) and five mRNAs (ATP2B4, AOX1, ALDH1A2, TSPAN1, and SLC43A1) in the normal and tumor tissues of PCa; right: Kaplan–Meier curves of prognostic models built with the expression of hsa-miR-222-3p, hsa-miR-221-5p, ATP2B4, AOX1, ALDH1A2, TSPAN1, and SLC43A1, respectively.

As illustrated in Figures 5, 6, all of the nodes in the core ceRNA network were capable of both distinguishing PCa tumor samples from normal samples and predicting patient disease-free survival. For instance, the lncRNA SNHG3, which is significantly up regulated in PCa tumor tissues (*t*-test *p*-value <2.2e-16, Figure 5D), exhibited a striking discrimination among tumors and normal samples as calculated by the AUC of the ROC curve (AUC = 0.92, Figure 5C). In addition, the log-rank test revealed that its up regulated expression was also associated with poor prognosis in patients with PCa (Figure 5G). Consistently, as shown in Figure 5J, the forest plot of hazard ratios indicated that SNHG3 is a risk factor for poor prognosis. However, all of the other RNAs in the core ceRNA network were protective factors (Figure 5J) whose high expression was associated with a good prognosis (Figures 5H,I, 6C–I). Additionally, most of these RNAs were significantly down regulated in PCa tumor tissues (Figurea 5H,I, 6C–G) and showed good performance in identifying tumor tissues (AUC > 0.80, Figures 5C, 6A,B). Interestingly, we also observed that two protective factors were significantly down regulated in PCa tumor tissues (Figures 6H–I), implying their changing functional role through PCa progression.

**FIGURE 7:**
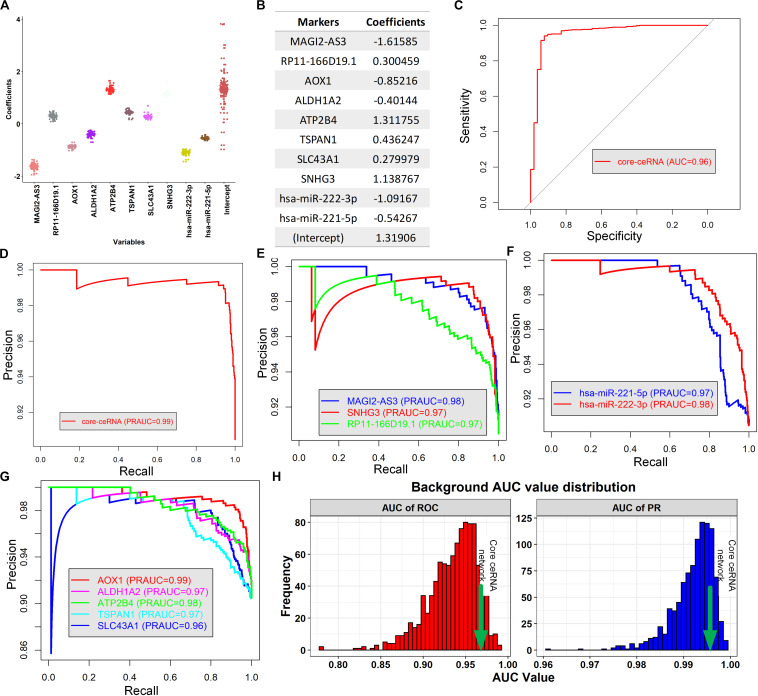
Building a novel diagnostic model and evaluate its performance. **(A)** The boxplot represents the distribution of each coefficient during the LOOCV procedure. **(B)** The coefficient of each ceRNA variable of the logistic model. **(C,D)** The AUC of ROC and PR curve of the logistic model for discriminating tumor tissue from normal controls, respectively. **(E–G)** The AUC of PR curve for discriminating the tumor and normal tissues based on the expression of lncRNAs, miRNAs and mRNAs in the core ceRNA network, respectively **(H)**.

Since each individual RNA in the core ceRNA network exhibited diagnostic and prognostic values, we investigated whether the combination of these factors could provide new opportunities for predicting PCa initiation and prognosis. First, a logistic regression model was constructed to discriminate the PCa tissues from normal controls in TCGA (Figures 7A,B). To avoid overfitting, the leave one out cross validation (LOOCV) was used to evaluate the model performance (Methods). We observed that the combination of 10 RNAs achieved ∼96% AUC of the ROC curve in excluding normal samples (Figure 7C), indicating the potential for early detection of PCa. We further assessed the AUC of PR to avoid the affection by unbalanced classes, which ends up with a consistent result that AUC value of PR achieved ∼99% (Figure 7D). In addition, the individual RNA in the ceRNA network also exhibited high AUC value of PR (>90%, Figures 7E–G). To further validate the diagnostic value of the core ceRNA network, we compared its performance with that obtained from randomly selected gene sets, serving as a background. As shown in Figure 7H, the AUC value of ROC and PR curve of the ceRNA network significantly better than did a random model (*p*-value < 0.01, *t*-test), suggesting it plays a role in early detection of PCa.

**FIGURE 8:**
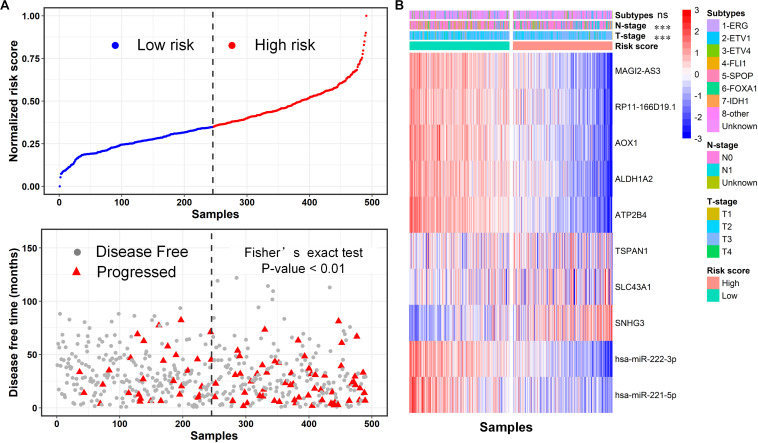
Evaluation of prognostic significance of the core ceRNA network using the risk score formal. **(A)** The distribution of disease-free survival data of the high-risk and low-risk subgroups for the TCGA-PRAD cohort. **(B)** The heatmap of the 12 RNA expression profiles for high-risk and low-risk subgroups, and the correlation between the risk score based subgroups and other subtypes including T staging, M staging and a molecular based subtype. ***p < 0.001; ns: non-significance.

We further defined a risk score to link the expression of these RNAs and the disease-free survival time using Cox-proportional hazards analysis according to a previous study (Van der Laan et al., 2003; Liu et al., 2018) (Methods). Then, we calculated the risk score for each PCa patient from TCGA and divided this population into high-risk and low-risk subgroups by the median value (Figure 8A, top). Remarkably, we observed that most of the progressed patients possessed high risk (*p*-value <0.01, Fisher extract test) compared to disease-free. Moreover, the heatmap of 10 RNA expression profiles revealed a distinct expression pattern for the two subgroups (Figure 8B), confirming the prognostic value of the ceRNA network. In addition, we investigated the association between the risk-score-based groups and other patients’ classifications, including molecular-based subtypes and tumor TNM staging. Fisher’s exact test showed that the risk score was significantly associated with tumor T staging and N staging rather than with the molecular subtype (Figure 8B), suggesting that the expression pattern of the core ceRNA network defined novel clinically meaningful groups. Taken together, these results indicate that the core ceRNA network exhibited high diagnostic and prognostic significance.

### PCa Patient Stratification Based on the Core ceRNA Network

**FIGURE 9:**
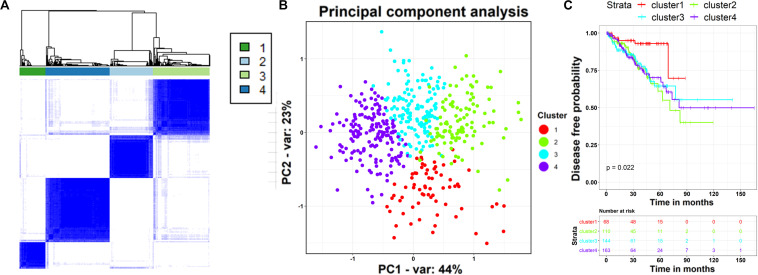
TCGA-PRAD patients stratification based on the expression of RNAs in the core ceRNA network. **(A)** Consensus clustering analysis of 492 TCGA-PRAD samples based on the expression profile of the RNAs in the ceRNA network. The heatmap shows the sample-by-sample Pearson’s correlation coefficient. **(B)** TCGA-PRAD populations identified after unsupervised clustering in panel **(A)**. Each point depicts a single patient, colored according to cluster designation. We reduced dimensionality by principal component analysis (PCA), and use the first and second principal components (*x*-axis and *y*-axis) to visualize the expression patterns of different clusters. **(C)** Kaplan–Meier curves and risk table showing the association between the ceRNA network-based subtypes and the disease free survival time.

As cancer is a complex and wildly heterogeneous disease, cancer informatics studies have raised fundamental questions regarding patient stratification using their molecular profiles (Wold et al., 1987). Because the above results have demonstrated the strikingly diagnostic and prognostic values of the core ceRNA network, we investigated whether the ceRNA network is capable of classifying the PCa population into clinically relevant subtypes. Presently, the expression profiles of the RNAs in the core ceRNA network were used as genomic signatures for patients with PCa from TCGA, and the unsupervised consensus clustering approach was subsequently applied to discover distinct subgroups. The results indicated that patients with PCa were subjected to four distinct clusters (Figure 9A) with different patient numbers (68, 110, 144, and 163, respectively). Subsequently, the separation between the different patient groups was further validated by the principal component analysis (Xin et al., 2009) (PCA) using the expression profile of the ceRNA network. Remarkably, the PCA map showed lower intra-cluster patient-to-patient similarity compared to the inter-cluster similarities (Figure 9B). What’s more, the first two principal components contributed up to 67% of the total variation, and patients in different subgroups exhibited distinct expression profile patterns. To assess whether the stratification determined by the core ceRNA network was associated with clinical outcomes, we evaluated the prognostic performance of the clusters with respect to disease-free survival using the Kaplan–Meier survival analysis. As displayed in Figure 9C, the ceRNA network-based subtypes were significantly associated with disease-free survival in this PCa population (*p* value <0.05, log-rank test). Taken together, these findings demonstrate that by using the nodes in the core ceRNA network as probes, we can identify novel patient subgroups with significant clinical outcomes.

## Discussion

Prior work has demonstrated the critical role of non-coding RNAs, such as miRNAs and lncRNAs, in regulating tumor onset and development. Huang et al., for example, reported that mir-210 is capable of regulating the hypoxic response of tumor cells and further controlling tumor growth (Li et al., 2017). mir-377 exhibited a suppression effect on esophageal cancer initiation and progression by inhibiting CD133 and VEGF (Liuqing et al., 2013). As for lncRNAs, increasing evidence has suggested their broad functional roles in mediating tumor formation and metastasis of many cancer types. For instance, lncRNAs PCAT8 and PCGEM1 are highly overexpressed in aggressive prostate cancer and strongly enhance androgen-receptor-mediated gene activation and proliferation in prostate cancer cells (Wei et al., 2017). Additionally, X-inactive specific transcript (XIST) promotes the tumor development by triggering miR−133a/EGFR signaling in Pancreatic Cancer (Falzone et al., 2019). Moreover, both lncRNAs and miRNAs are able to serve as promising diagnostic and prognostic biomarkers across multiple tumor types, such as oral cancer (Guo et al., 2015) and ovarian cancer (Hirohashi and Kanai, 2010). Given the functional interdependencies between the distinct RNA molecules, cancer pathology studies have shifted their focus from individual RNAs that carry cancer-associated dysregulation toward a network-based perspective of underlying mechanisms such as the ceRNA network hypothesis (Salmena et al., 2011). For example, Wang et al. recently established a mRNA-miRNA-lncRNA network, which was demonstrated to be associated with the prognosis of pancreatic cancer (Wang et al., 2019). Additionally, the ceRNA network was reported to exhibit a strong relationship with certain clinical features in human head and neck squamous cell carcinoma (Fang et al., 2018). Specifically, for prostate cancer, Liu et al. systematically investigated the gain and loss of ceRNAs and suggested its potential for development biomarkers and therapeutics (Liu et al., 2016). Nevertheless, comprehensively elucidating the diagnostic and prognostic significance of the ceRNA network in PCa is still insufficient. In this study, using the expression data from multiple independent PCa-related studies, we constructed a core ceRNA network with high confidence by employing three lncRNA-gene prediction approaches and key node filter strategies and then established a logistic model and risk score formula to evaluate its diagnostic and prognostic value, respectively. We found that the combination of expression of 10 RNAs not only achieved an AUC of ROC and PR curve of more than 95% in excluding normal prostate samples but also can be used to predict the patient’s prognosis as well as divide patients into clinically relevant subtypes.

In the present study, we used the common mRNAs and miRNAs from multiple GEO and TCGA studies, and identified the ceRNAs with diagnostic significance. As can be observed in Figures 1, 2, if we only used the TCGA dataset, it seems that we can obtain more candidate ceRNAs for further analysis such as identification of diagnostic and prognostic biomarkers. However, it should be noted that the most critical reason that limits the broad clinical adoption of RNA expression profiles is the poor reproducibility across biological replicates due to technical and biological noise (Alvarez et al., 2016). For example, one RNA-seq dataset (GSE89223) obtained from GEO was based on the Ion Torrent Proton platform, while RNA-seq data obtained from TCGA was generated with the Illumina HiSeq platform. What’s more, samples of these two datasets are collected from different countries/regions. Thus, there could be a risk that a part of ceRNAs in the TCGA dataset may be wrongly selected as diagnostic and prognostic biomarkers due to the sample or platform induced data bias. To address this problem, we integrated the differentially expressed RNAs derived from multiple independent PCa-related studies using different profiling platforms. Only the differentially expressed RNAs that commonly appeared in different studies were selected for further analysis to reduce the risk of data bias and make our analysis more reliable. Non-coding RNAs usually exert specific functions through protein-coding RNAs. Thus, the shared genes were adopted for GOBP and KEGG enrichment analyses, which have been widely used for investigating the underlying mechanism. The enrichment analysis revealed that deregulated mRNAs were significantly enriched in PCa initiation- and progression-related biological processes, including metabolic (Liu, 2006; Tennant et al., 2010) and cell adhesion-related processes (Chen et al., 2015). Furthermore, enrichment in several signaling pathways, such as the wnt signaling pathway (Wasilenko et al., 1997) and calcium signaling pathway (Albert-László et al., 2011), may provide insight into the upstream molecular mechanism for PCa development. Thus, these differentially expressed RNAs must be potential biomarkers for diagnosis and prognosis.

Inferring context-specific ceRNA interactions with high confidence is crucial for better understanding ceRNA regulatory mechanisms and their biological significance. In the present study, the ceRNA network was constructed with “GDCRNATools” (Li R. et al., 2018) based on three strategies, including the significance of the hypergeometric test, Pearson correlation analysis and regulation pattern analysis. Specifically, the hypergeometric test was used to test whether the number of shared miRNAs by a lncRNA and gene is significant, ensuring the reliability of the inferred lncRNA-mRNA pairs. Pearson correlation analysis and regulation pattern analysis were applied to ensure that the regulatory associations between the lncRNAs and genes were tissue-specific interactions in PCa.

This ceRNA network thereby provides a tissue-specific primary regulatory landscape among the endogenous RNAs. Nevertheless, as it consists of numerous compounds and interactions among them, it is difficult to elucidate the main underlying mechanisms of PCa. We therefore adopted three criteria to filter the key nodes that are associated with PCa onset and progression and subsequently specify the core ceRNA network architecture, which may increase the opportunity for PCa early detection and enhance the reliability of prognosis monitoring. As a result, the final core ceRNA network consists of 3 lncRNAs, 2 miRNAs, 5 mRNAs and 16 interactions among them. All of these RNAs are capable of discriminating the tumor samples and normal tissues with high accuracy, indicating their diagnostic value. In the core ceRNA network, only 3 of 10 RNAs (SNHG3, TSNAP1 and SLC43A1) are up regulated in the tumor tissues, suggesting their potential tumor promotion effect. However, the other RNAs are tumor-suppressor RNAs whose low expression is associated with an unfavorable prognosis. Intriguingly, we found that the high expression of two potential tumor promotor genes (TSNAP1 and SLC43A1) is associated with a better prognosis in PCa. This seeming contradiction can be explained in part by the alternation of their functional roles during PCa development. Notably, most of these RNAs have been well investigated in PCa, indicating the critical role of this core ceRNA network.

One of the most important findings of this study is that using the combination of different RNAs as candidate diagnostic biomarkers of PCa achieved an AUC of ROC and PR curve of ∼96 and 99%, respectively. To avoid overfitting, the LOOCV was applied to evaluate the performance of the combination of the ceRNAs. This result supported the diagnostic significance of the ceRNA network in PCa detection, implying that PCa initiation is a complicated process involving multiple types of RNAs. Moreover, this study provides a novel strategy for developing diagnostic models with high accuracy. However, we have not generalized our conclusion to other tumor types. Thus, the diagnostic value of the ceRNA network on other tumor types needs further study. By constructing a risk score formula according to a previous description, the TCGA-PRAD cohort can be divided into low- and high-risk score subgroups. Remarkably, the high-risk score is significantly associated with poor disease-free survival, indicating that the risk score is a new risk factor for patient prognosis. Additionally, the risk score-based subgroup is significantly associated with tumor T staging and N staging. This could be because of the significant association between TNM staging and patient prognosis. However, it is not associated with a well-established molecular subtype (Abeshouse et al., 2015), suggesting that the ceRNA network must have defined a novel patient stratification. Strikingly, using an unsupervised clustering approach, the TCGA-PRAD cohort was stratified into four clinically relevant clusters with distinct molecular profiles defined by ceRNA expression, which further proves the prognostic value of the ceRNA network. This is the first study to provide core ceRNA network-based stratification of PCa patients, highlighting a feasible way to improve patient stratification in the future.

In conclusion, we have integrated the RNA expression profiles derived from multiple independent PCa-related studies to increase reliability of data, and subsequently constructed a high confident PCa-specific core ceRNA network by employing three lncRNA-gene inference approaches. The diagnostic and prognostic significance of the core ceRNA network were then validated in PCa patient cohort. However, despite our encouraging results, some limitations are worth noting. For the prognosis evaluation of the ceRNA network, we only assessed the association between the ceRNA network and disease-free survival rather than the overall survival due to the limitation of the clinical data. In addition, our research mainly focuses on evaluation of the prognostic significance of ceRNA networks in PCa patients with bioinformatics tools, and future work focusing on the *in vitro* and *in vivo* validation before clinical application is still needed. However, we have reason to believe that in the not-too-distant future, along with the development of various experimental approach, the main findings of this research will be validated *in vitro* or *in vivo*, and may be directly used for clinical purposes. Nevertheless, our findings coupled early tumor detection and prognostic prediction, which are usually described as two seemingly disconnected entities, with the refined core ceRNA network. This study also provided some key molecular clues regarding tumor onset and progression, which might be useful for further investigation of PCa.

## Data Availability Statement

miRNA expression profiles of two independent, PCa-related studies were downloaded from the Gene Expression Omnibus (GEO) database (GSE76260 and GSE21036). As for the pcmRNA and lncRNA, two RNA-seq datasets derived from independent PCa-related studies were obtained from GEO. The GEO accession numbers of the two datasets are GSE89223 and GSE104131.

## Author Contributions

YW, JW, and WX designed the study. ZG, LH, and JW wrote the manuscript and performed *in silico* analysis of the data. YF and YM collected some data. ZG, LH, YF, and ZWu revised the manuscript. YF, ZWu, YM, YL, SL, HW, LJ, ZWa, and FL modified the language. All authors have read and approved the final manuscript.

## Conflict of Interest

The authors declare that the research was conducted in the absence of any commercial or financial relationships that could be construed as a potential conflict of interest.

## Footnotes

1 https://cghub.ucsc.edu

2 http://www.gencodegenes.org
